# A Remedy for Lumbago

**Published:** 1887-11

**Authors:** 


**A Remedy for Lumbago.**—According to an exchange, Prof. Burrgraeve recommends the application of a mixture of equal parts of collodion, tincture of iodine, and ammonia-water, applied widely over the affected region with a camel's hair brush, as an "instantaneous" remedy for lumbago.—*N. Y. Medical Journal.*

